# Nanomedicine Strategies for Overcoming Temozolomide Resistance in Glioblastoma

**DOI:** 10.2174/011570159X441441260318061159

**Published:** 2026-05-15

**Authors:** Yancheng Lv, Jiazu Sa, Ke Wang, Jie Zhou

**Affiliations:** 1 Department of Neurosurgery, The Affiliated Hospital, Southwest Medical University, Luzhou, 646000, PR China;; 2 Sichuan Clinical Medical Research Center for Neurosurgery, Luzhou, 646000, PR China;; 3 Neurological Diseases and Brain Function Laboratory, The Affiliated Hospital, Southwest Medical University, Luzhou, 646000, PR China;; 4 The Affiliated Hospital, Southwest Medical University, Luzhou, 646000, PR China;; 5 Key Laboratory of Advanced Technologies of Materials, Ministry of Education, School of Materials Science and Engineering, Southwest Jiaotong University, Chengdu, 610031, China;; 6 Institute of Biomedical Engineering, College of Medicine, Southwest Jiaotong University, Chengdu, 610031, P. R. China

**Keywords:** Drug resistance, glioblastoma, nanoparticle drug delivery system, temozolomide, glioblastoma multiforme, GBM therapy

## Abstract

**Background:**

Glioblastoma multiforme (GBM) is among the deadliest of brain tumors. While temozolomide (TMZ) is the standard treatment, tumors quickly develop resistance, leading to patient relapse. The blood-brain barrier (BBB) and complex tumor microenvironments further limit drug efficacy.

**Methods:**

We reviewed literature from 2015 to 2024 *via * PubMed and Web of Science, focusing on nanoparticle-based strategies designed to overcome TMZ resistance. We specifically analyzed how these carriers tackle mechanisms like MGMT overexpression and drug efflux.

**Results:**

Emerging nanomedicines-including liposomes, polymeric nanoparticles, and inorganic carriers-show promise in preclinical models. Key advances include co-delivering TMZ with MGMT inhibitors or siRNAs to re-sensitize tumor cells. Stimuli-responsive systems that release drugs only within the tumor acidity or hypoxia also reduce systemic toxicity.

**Conclusion:**

While nanotechnology offers tools to bypass biological barriers and reverse resistance, clinical translation remains slow. Future success depends on solving manufacturing scalability, ensuring long-term safety, and navigating regulatory pathways. Integrating these tools with personalized molecular profiling represents the next step in GBM therapy.

## 1 INTRODUCTION

Glioblastoma multiforme (GBM) is the most aggressive primary brain malignancy, presenting a formidable clinical challenge due to its intrinsic therapeutic resistance and rapid recurrence. According to the 2017-2021 Central Brain Tumor Registry of the United States (CBTRUS) report [1]. GBM accounts for 13.9% of all primary and 51.5% of malignant brain tumors, with a notably higher incidence in males. Despite multimodal treatments involving surgical resection, radiation, and temozolomide (TMZ) chemotherapy, median patient survival remains poor, rarely exceeding 15 months [2-5].

TMZ is an alkylating agent that exerts antitumor effects primarily through DNA methylation, establishing it as the standard first-line therapy. However, more than 50% of patients develop intrinsic or acquired resistance. This resistance is driven by multifactorial mechanisms, including O6-methylguanine-DNA methyltransferase (MGMT) overexpression, dysregulated DNA damage response pathways, and immunosuppression within the tumor microenvironment (TME) [6-9]. Consequently, developing innovative strategies to restore TMZ sensitivity while minimizing systemic toxicity is urgent.

Recent studies (2021-2024) confirm that combining epigenetic drugs (*e.g*., the HDAC inhibitor belinostat) with radiotherapy improves 6-month progression-free survival in newly diagnosed GBM to 84% (vs. 54% in controls) and extends median overall survival by approximately 2.7 months [10, 11]. Concurrently, emerging strategies-including CRISPR/dCas9 epigenetic editing, nanodelivery systems, and multi-omics AI integration-show significant potential for tumor suppression and reversing drug resistance. These advances provide a robust empirical foundation for precision GBM therapy [12]. Although TMZ remains the first-line standard, its efficacy is severely limited by intrinsic and acquired resistance. Key mechanisms include enhanced drug efflux, reduced uptake, and target mutation [13]. Elucidating these mechanisms, particularly epigenetic remodeling pathways, is crucial for developing novel therapeutic tools to overcome this resistance bottleneck [14-18]. Notably, nanotechnology offers transformative solutions to these challenges. Engineered nanocarriers (*e.g*., lipid nanoparticles, polymeric micelles) significantly enhance drug bioavailability and facilitate blood-brain barrier (BBB) penetration [19]. By co-delivering TMZ with sensitizers like MGMT inhibitors, these platforms achieve spatiotemporally controlled release and synergistically disrupt resistance pathways while minimizing systemic toxicity [20-23]. In addition, targeting GBM-specific biomarkers (*e.g*. EGFRvIII or IL-13Rα2) using surface ligand functionalization strategies has effectively enhanced tumour selective accumulation in preclinical studies [24]. Collectively, these multidisciplinary innovations underpin the development of precision treatments designed to circumvent GBM drug resistance [25-28].

Despite this progress, clinical translation faces hurdles, including limited tumor penetration due to heterogeneity, premature drug leakage, and immune clearance [29, 30]. Biomimetic and stimulus-responsive systems (*e.g*., pH- or enzyme-triggered release) have emerged as promising strategies to address these limitations. Furthermore, integrating nanotechnology with immunotherapies or gene editing offers a multidimensional approach to re-sensitize resistant tumors. It is acknowledged that second-line agents (*e.g*., lomustine, bevacizumab) and other glioma subtypes (*e.g*., oligodendrogliomas) share certain therapeutic challenges [31, 32]. However, given that TMZ remains the cornerstone of standard-of-care and GBM represents the most aggressive and drug-resistant phenotype with distinct blood-brain barrier characteristics, this review prioritizes strategies to restore TMZ sensitivity in GBM specifically [33]. This review summarizes recent nanomedicine advances in targeting TMZ-resistant GBM. By bridging nanomaterial design with clinical needs, we explore how next-generation platforms can revolutionize the therapeutic landscape.

Unlike previous reviews that broadly discuss brain tumor nanomedicine, this article specifically maps distinct nanocarriers to the molecular "resistance network" of GBM. We not only summarize recent efficacy data but also critically analyze why-despite promising lab results-these technologies struggle to reach clinical trials, focusing on manufacturing and regulatory hurdles (Fig. **1**).

### 1.1 Review Methodology

We searched databases including PubMed, Scopus, and Web of Science for studies published primarily between January 2015 and December 2024. Search terms included "glioblastoma," "temozolomide resistance," "nanoparticles," "BBB penetration," and "clinical translation." We prioritized studies that provided mechanistic insights into reversing drug resistance rather than just reporting cytotoxicity.

## 2 MECHANISMS OF GBM RESISTANCE TO TMZ

### 2.1 Mechanisms of TMZ Action in GBM Treatment

A recent umbrella review confirms that TMZ, as the standard-of-care chemotherapeutic for gliomas, significantly prolongs overall survival (OS) and progression-free survival (PFS) when combined with radiotherapy or molecularly targeted agents. Notably, specific combinations reduced the PFS hazard ratio to 0.60 even in patients with unmethylated MGMT promoters, reinforcing TMZ's central role in multimodal precision therapy [34]. However, pharmacokinetic limitations persist. Recent molecular dynamics simulations indicate that TMZ faces a high transmembrane free energy barrier (~50 kcal/mol) at physiological temperatures, hindering passive diffusion across brain cell membranes [35]. This barrier, primarily attributed to hydrogen bonding with membrane cholesterol and cerebrosides, necessitates the optimization of molecular structures or the development of advanced delivery systems to enhance brain accumulation.

TMZ is an imidazotetrazine prodrug lacking intrinsic antitumor activity [36]. Upon systemic administration, it undergoes spontaneous hydrolysis to the active metabolite MTIC, which further degrades into AIC and the methyldiazonium cation [37]. This cation methylates DNA purine bases-particularly at the O6-guanine and N7-guanine positions-causing DNA double-strand breaks and cell cycle arrest that trigger apoptosis in the absence of effective repair [7]. However, therapeutic efficacy is severely compromised in resistant GBM. Recent studies reveal that resistance involves not only MGMT promoter demethylation but also genome-wide methylation remodeling, including hypermethylation of the MGMT gene body and epigenetic alterations in key signaling pathways (*e.g*., MEF2B hypermethylation, MAPK4/6 hypomethylation) [38, 39].These epigenetic shifts collectively drive tumor infiltration and survival, neutralizing the cytotoxic impact of TMZ.

### 2.2 Resistance Mechanisms for TMZ

#### 2.2.1 Enhancement of DNA Repair Mechanisms

The overexpression of DNA repair-related genes in glioma cells enhances resistance to TMZ. Among these, O6-methylguanine-DNA methyltransferase (MGMT) is the most notable, functioning *via* a "suicide enzyme" mechanism to remove TMZ-induced methyl groups from DNA. Specifically, MGMT transfers the methyl group from the O6 position of guanine to its own cysteine residue, effectively repairing DNA damage and protecting tumor cells from cytotoxicity [12, 40]. While TMZ exerts its effects mainly through DNA alkylation, its therapeutic efficacy is significantly diminished by the upregulation of multiple DNA damage repair (DDR) mechanisms in GBM cells. Beyond MGMT, other pathways-including base excision repair (BER), mismatch repair (MMR), nucleotide excision repair (NER), and homologous recombination (HR)-contribute significantly to TMZ resistance. Although several drugs targeting DDR have been developed, their clinical efficacy remains unsatisfactory [41]. Recently, post-translational modifications (PTMs) have emerged as central regulators of genomic stability, influencing MGMT-mediated repair, MMR, BER, and double-strand break repair [42]. Targeting the enzymes responsible for these PTMs offers a strategy to disrupt DDR signaling and enhance TMZ cytotoxicity [43]. Integrating these preclinical findings with clinical realities highlights the potential of modulating PTM-driven networks as a novel therapeutic strategy to overcome resistance [44].

#### 2.2.2 Increased TMZ Efflux

The ATP-binding cassette (ABC) transporter family plays a central role in GBM drug resistance. High expression of transporters such as ABCB1 (P-gp), ABCC1 (MRP1), and ABCG2 (BCRP) in both GBM cells and the blood-brain barrier (BBB) promotes the efflux of chemotherapeutics, including TMZ [45]. This efflux reduces intracellular drug concentrations, impairing therapeutic efficacy and accelerating the development of a resistant phenotype. A qPCR analysis of 159 GBM samples revealed that ABCC1, ABCC3, and ABCG2 levels were significantly elevated in both primary and recurrent tumors compared to non-tumor tissue; notably, high ABCC1 expression correlated strongly with shorter progression-free survival (PFS) [46]. Furthermore, TMZ acts as both a substrate and an inducer of ABC transporters; TMZ exposure upregulates ABCB1 expression, creating a positive feedback loop that drives acquired resistance. While inhibiting transporters like ABCB1 and ABCB5 enhances TMZ accumulation and apoptosis, these mechanisms involve complex networks encompassing PI3K/Akt/NF-κB signaling, miRNA regulation, and stem cell properties [47]. However, due to functional redundancy, single inhibitors often fail. Future strategies must prioritize multi-target inhibition, signaling pathway regulation, and gene editing. Additionally, fatty acids (*e.g*., PUFA) and novel small-molecule D2 inhibitors have shown promise in enhancing intracellular TMZ concentrations by modulating transporter activity [48, 49]. In conclusion, targeting the ABC family holds promise for enhancing TMZ efficacy. Nevertheless, clinical translation remains challenged by functional redundancy, toxicity concerns, and the complexity of the resistance network.

#### 2.2.3 Role of Glioma Stem Cells (GSCs)

GSCs possess unique properties, such as self-renewal and multidirectional differentiation, which confer resistance to conventional radiotherapy and enable rapid post-treatment regeneration [50, 51]. These characteristics define GSC "stemness." Recent studies identified cell communication network factor 1 (CCN1), highly expressed in GSCs, as a key regulator of this stemness, proliferation, and mesenchymal transition [52]. Furthermore, an analysis of CD133+ stem cells revealed 126 differentially expressed lncRNAs; notably, LOXL1-AS1 was upregulated while PAX8-AS1 was downregulated, providing potential therapeutic targets [52, 53]. Additionally, GSCs maintain a tumor-tumor connectivity (TTC) network *via* nanotubes and tumor microtubes (TMs), which are critical for morphology and chemoresistance. Adenylate cyclase-associated protein 3 (ADD3) has been identified as a key morphological regulator essential for TM formation [54].

#### 2.2.4 Tumor Immune Microenvironment (TME)

First, single-cell multi-omics and spatial transcriptomics reveal that GBM stem cells exhibit high phenotypic plasticity. These cells dynamically adapt to TME signals to evade immune clearance and drive recurrence [50]. Second, immune cells (*e.g*., M2 macrophages, Tregs), astrocytes, and vascular endothelial cells within the TME support GSC survival and induce immune escape by secreting various cytokines, significantly impairing the efficacy of drugs like TMZ [55]. Novel agents like the SMAC mimetic Xevinapant can induce GSC apoptosis and remodel the TME to reverse immunosuppression. However, long-term treatment may still lead to adaptive escape *via* phenotypic transformation and resistance gene expression [56].Consequently, dual-targeting strategies focusing on both "stem cells and the microenvironment" offer a solid theoretical basis for developing combined immunotherapies [57].

#### 2.2.5 Formation of the "Tumor Drug Resistance Network"

Recent studies identify tumor microtubules (TMs) as membrane protrusions that facilitate intercellular communication and form a therapeutic resistance network [58, 59]. TMs enable the transfer of calcium ions, mitochondria, and signaling molecules [60]. Alterations in intercellular calcium waves (ICWs) synchronize tumor cell responses, creating a "toxicity equalization" effect that diminishes chemotherapeutic efficacy [61-64]. Connexin-43 (Cx43) gap junctions connect these TMs; inhibiting Cx43-*via* gene knockdown or Tonabersat combined with TMZ disrupts this network and enhances drug sensitivity [65, 66]. Additionally, growth-associated protein 43 (GAP-43) is integral to TM formation. Knocking down GAP-43 reduces invasion and chemoresistance. Knockdown of GAP-43 reduces glioblastoma invasion, migration, chemoresistance, and tumor self-repair capabilities [58, 67-69]. In mouse models simulating post-treatment conditions, TMs were observed delivering nuclei to dead cells, regenerating resistant populations; notably, this was limited by GAP-43 knockdown [67, 70]. Recognizing TMs as a therapeutic target, recent research identified a novel microtubule-targeting agent (MTA), AUS_001, which effectively inhibits GSC growth, crosses the blood-brain barrier, and counteracts chemotherapeutic resistance [71].

It is critical to recognize that in a clinical setting, these resistance mechanisms do not act in isolation. A single patient's tumor may simultaneously exhibit MGMT overexpression, enhanced efflux pump activity, and metabolic remodeling. This intrinsic heterogeneity explains why single-target interventions often fail and highlights the absolute necessity for multifunctional nanoplatforms that can attack multiple pathways at once.

### 2.3 Expansion of Intrinsic Resistance Mechanisms in Tumour Cells

In recent years, research on glioblastoma (GBM) resistance to temozolomide (TMZ) has been breaking through, gradually upgrading from a single-point understanding of molecules and signalling pathways to a complex mechanism that is multi-pathway, multi-level networked and tumour microenvironment-dependent. The high expression of molecules such as the DNA repair enzyme MGMT, the methylation enzyme DNMT3B, and the drug efflux protein ABCB1 certainly provides direct material for the underlying drug resistance support [72, 73]. However, epigenetic and signalling pathway interactions have now become central hubs in the regulation of drug resistance. DNMT3B is activated by the PI3K/Akt upstream signalling axis, which enhances DNA methylation levels and TMZ resistance, and down-regulation of this axis molecule significantly reverses drug resistance [74]. Aberrant activation of STAT3-MGMT signalling also contributes to drug resistance, whereas Siramesine enhances apoptosis sensing by down-regulating this pathway, exerting a synergistic reversal effect [75].

The signal transduction network is highly complex, with pathways such as AKT-mTOR, PI3K/Akt, HIF-1α/2α, Wnt/ β-catenin, NF-κB, *etc*., crosstalking or cross-talking to regulate cell proliferation, apoptosis, and metabolic remodelling, and are tightly cross-linked with epigenetic, autophagy, and death signals [72]. Typical examples include activation of the HIF-1α/2α-IGF1R axis in a hypoxic microenvironment, elevating TMZ resistance and inducing tumour infiltration [76]. Peiying Bai *et al*. Activation of NF-κB by ECM molecules is involved in tumour-promoting feedback, exacerbating drug resistance and invasiveness [76]. In addition, quercetin, a variety of natural products, can synergistically down-regulate *MGMT* and increase sensitivity through multiple signalling targets [77].

Novel cell death pathways are a "gateway" for drug resistance reversal. Iron death, apoptosis, autophagy and other pathways have been demonstrated to be bidirectionally regulated in refractory GBM-resistant cells. IRP1 induces iron death by regulating the LCN2/FPN1 signalling axis, and experimental up-regulation of IRP1 reverses TMZ resistance and promotes tumour cell killing [78]; Muscone restores the susceptibility of TMZ-resistant cells to apoptosis through the TOP2A/THBS2 mechanism, opening a window for novel combination therapy [78]. Other natural small molecules (*e.g*., thymoquinone, essential oil of Ligusticum chuanxiong, *etc*.) can inhibit the expression of *AKT* and *MGMT*, and up-regulate apoptosis and autophagy, thus achieving a synergistic effect in the reversal of drug resistance [73, 79].

Changes in the tumour microenvironment are increasingly seen as one of the sources of drug resistance. Suppressive microenvironments constructed by immune cells (*e.g*., MES-MDM, CD8+ T-cell infiltration, bone marrow-derived immune cell mobilisation) can dramatically reduce the efficacy of TMZ and immunotherapy [80, 81]. MES-MDM secretes TNF and activates the CELSR2/p65/Kla-HDAC1/EPAS1 axis, which induces tumour phenotypic transformation and drug resistance, and the combination of TREM1 inhibitor with TMZ and immune checkpoint inhibitors significantly enhances the efficacy of drug-resistant GBM models [82].

ECM remodelling is an emerging key to tumour drug resistance. P4HA1 hydroxylation *via* YAP drives COL1A1 transcription and collagen deposition to create a favourable drug resistance microenvironment, and inhibition of this axis increases TMZ sensitivity and prolongs animal survival [83]. Tumour cell heterogeneity and phenotypic plasticity (*e.g*., MSN-mediated PN-MES transformation) similarly reinforce drug resistance, and GNE-317 inhibitors interrupt this transformation and enhance TMZ and radiotherapy effects [84].

From a comprehensive perspective, the resistance mechanism of GBM to TMZ is driven by the complex coupling of "multi-molecule-multi-pathway-microenvironment-cell phenotype". With the application of advanced technologies such as multi-omics and spatial genomics, the study of drug resistance mechanisms has been upgraded from the traditional "single molecule and single pathway" mode to a networked and individualised panoramic mechanism analysis. Specifically, tumour cells' own escape mechanisms (*e.g*., MGMT-mediated DNA repair, epigenetic remodelling involving STAT3 and DNMT3B, activation of pro-survival pathways such as Wnt/β-catenin and PI3K/Akt), microenvironmentally-driven stress adaptation (including hypoxia-induced metabolic reprogramming, infiltration of immune-suppressive cell subsets such as mesenchymal macrophages and collagen deposition), and tumour stem cell-associated heterogeneous evolution, combine to regulate the expression of drug-resistant phenotypes.

In this multilevel and multifactorial dynamic network, single-target therapeutic strategies are difficult to achieve a durable reversal of drug-resistant phenotypes, while traditional systemic drug delivery faces the risk of toxicity due to low blood-brain barrier (BBB) penetration and systemic non-specific distribution. In recent years, the rapid development of nanomedicine has brought breakthrough ideas to address these challenges - through rationally designed multifunctional delivery systems, integrating targeting ligands, environment-responsive modules, and drug synergistic loading, it is possible to achieve precise spatial and temporal enrichment of drugs in tumour tissues and synergistic intervention of multiple pathways, greatly enhancing the efficacy of treatment (Fig. **2**).

Looking ahead, iron death, immune/autophagy synergy, signalling pathway interaction, tumour microenvironment remodelling, and multi-targeted natural product co-intervention will become the focus of breakthroughs in drug-resistant mechanisms and precision individualised therapy. Stratification and typing supported by multi-omics big data to achieve co-targeting and molecular individualisation will provide a new growth point for the clinical benefit of drug-resistant GBM patients. Finally, the spatiotemporal heterogeneity of GBM adds another layer of complexity. Resistance mechanisms are not static; they evolve under therapeutic pressure. A tumor recurrence may display a completely different resistance profile compared to the primary tumor, and cells in the hypoxic core may rely on different survival pathways than those at the invasive edge. This dynamic evolution renders 'static' therapeutic strategies ineffective over time.

## 3 OVERCOMING THE FIRST BARRIER: PENETRATING THE BBB

### 3.1 The Role of the BBB in TMZ Resistance Development in GBM

The BBB is a dynamic physiological barrier centered on brain microvascular endothelial cells (BMECs) [85], regulated by three key mechanisms. First, tight junction complexes (involving occludin, claudin-5, and ZO-1/ZO-2) strictly limit the passive diffusion of hydrophilic molecules larger than 500 Da [86]. Second, active efflux mechanisms rely on high expression of ATP-binding cassette (ABC) transporters-such as P-glycoprotein (P-gp), BCRP, and MRPs-on endothelial cells [87-89]. These transporters actively pump small-molecule drugs like TMZ back into the bloodstream, drastically reducing intracerebral concentrations. Third, the neurovascular unit (NVU) exerts dynamic control; astrocytes and pericytes modulate BMEC permeability *via* paracrine signaling (*e.g*., VEGF, ANGPTL4), introducing pathology-dependent variability in drug delivery [90].

Although TMZ is the standard chemotherapeutic for GBM, its efficacy is compromised by these barriers [91]. Despite favorable theoretical properties (194.15 Da; logP = -0.1), TMZ's passive diffusion is limited in practice, with brain concentrations reaching only 20-30% of plasma levels due to efflux activity. Furthermore, abnormal angiogenesis creates a heterogeneous, "leaky" barrier that paradoxically restricts drug distribution to invasive cells at the tumor margin, thereby protecting them from cytotoxic attack [92-94]. These findings identify efflux transporters as a primary bottleneck and highlight the need for novel strategies to enhance BBB penetration.

The first mechanism is microenvironment-driven resistance, in which glioma stem cells (GSCs) near the BBB are stimulated to maintain a chemoresistant phenotype through Notch and Wnt signaling pathway ligands secreted by endothelial cells [67]. Simultaneously, the high expression of P-gp and BCRP in tumor cells forms a "dual efflux pump" with BBB endothelial cells. This synergistic expression of efflux proteins and tumor barriers further diminishes TMZ accumulation in tumor cells [95].

The second mechanism involves TMs, which establish persistent, long-distance intercellular connections in GBM cells, forming a functional electrochemical network (*e.g*., voltage-gated calcium channels). This network enables the dispersion of damage signals (*e.g*., TMZ-induced DNA damage) across the entire tumor cell population [96]. Combined with the physical barrier of the BBB, this networked resistance mechanism protects drug-resistant subpopulations of tumor cells. Finally, the acidic tumor microenvironment and endothelial ionic gradients reduce TMZ lipid solubility and transmembrane efficiency, preventing the drug from reaching the threshold concentrations required to suppress MGMT expression.

### 3.2 Nanotechnology Advances BBB Delivery

BBB, while serving as a crucial physiological protector, poses significant challenges for therapeutic drug delivery targeting neurodegenerative disorders (*e.g*., Alzheimer's disease, Parkinson's disease) and cerebral malignancies (*e.g*., glioblastoma). However, recent advancements in nanotechnology have revolutionized BBB penetration strategies, offering promising solutions to this long-standing biomedical challenge [97-99]. The remarkable efficacy of nanomaterials in BBB penetration primarily stems from their distinctive physicochemical characteristics. Foremost among these is their nanoscale dimension, with optimal diameters below 200 nm, which facilitates transport through either endothelial gaps or cytosolic pathways of the BBB [100, 101]. Furthermore, surface charge engineering has demonstrated significant potential, as cationic nanoparticles exhibit enhanced interactions with the negatively charged BBB surface, thereby improving penetration efficiency [102, 103]. Complementing these approaches, the development of deformable liposomes with exceptional structural flexibility has shown remarkable adaptability to the dynamic architecture of the BBB, further augmenting drug delivery efficiency [104] (Fig. **3**).

Beyond conventional nanocarriers, the integration of Focused Ultrasound (FUS) with Microbubbles (MBs) offers a novel strategy for CNS drug delivery. This technique induces a localized, reversible opening of BBB tight junctions, facilitating targeted nanoparticle accumulation [105]. Recent studies demonstrate its ability to disrupt both the BBB and the Blood-Tumor Barrier (BTB) while modulating the immunosuppressive microenvironment in intracranial melanoma. Specifically, FUS/MBs trigger pro-inflammatory gene expression (*e.g*., TNF, IL-6) and enhance dendritic cell maturation (evidenced by increased CD86) within the tumor [106]. However, as immunostimulatory effects remain limited, combining this approach with immunotherapy or adjuvants may offer synergistic benefits. Transnasal drug delivery has also gained attention as a non-invasive alternative. By exploiting direct olfactory and trigeminal pathways, this method enables rapid CNS delivery while bypassing systemic metabolism and the BBB. Recently, integrating transnasal delivery with image guidance and FUS has significantly improved targeting precision and efficacy [107, 108].

Surface functionalization represents a critical strategy for targeted delivery. Organic nanoparticles, such as liposomes and polymers, can be engineered with ligands (*e.g*., lactoferrin or transferrin receptor antibodies) that target endothelial receptors (TfR, LRP1), thereby activating receptor-mediated transcytosis (RMT) [109]. For instance, zein nanoparticles chemically modified with polyethylene glycol (PEG) and Angiopep-2 (ANG2) were developed to deliver docetaxel (DTX); this design significantly enhances BBB permeability and exerts potent anti-glioblastoma activity [110]. Similarly, transferrin-modified chitosan nanoparticles have been engineered for transnasal delivery to achieve dual functionality: efficient transport across the nasal epithelium and specific uptake by glioblastoma cells [111]. Despite these advantages, transnasal systems face persistent challenges, including limited absorption surface area, restricted dosing capacity, rapid mucociliary clearance, and poor bioavailability of polar macromolecules [111].

### 3.3 Nanoplatform-Driven Multimodal Synergistic Therapy

Multimodal Synergistic therapy has emerged as a pivotal strategy in oncology, synergistically integrating diverse therapeutic modalities to amplify antitumor efficacy while minimizing systemic toxicity [112]. This paradigm represents the forefront of future tumor treatment development, achieving dual therapeutic optimization through enhanced drug targeting precision and elevated tumoricidal efficacy, coupled with significant reduction of off-target toxicity in healthy tissues and organs.

Multimodal nanotherapy has significantly advanced GBM treatment. For example, lithium phthalocyanine-based dendrimer nanoparticles (LiPTD NPs) have been developed to combine chemotherapy with sonodynamic therapy (SDT) [113]. These nanoparticles achieve 87.6% pH/temperature-responsive doxorubicin (DOX) release within 24 hours while activating the sonosensitizer chlorin e6 (Ce6) under ultrasound [112]. The resulting reactive oxygen species (ROS) induce potent apoptosis and necrosis, improving outcomes in GBM models [114]. Nanozymes have also emerged as effective SDT adjuvants, offering superior catalytic stability (>90% activity after 30 days) and cost-effectiveness (production costs reduced by 60-75%) compared to natural enzymes [115]. They potentiate SDT by catalyzing ROS generation (2.3-fold increase) and remodeling the tumor extracellular matrix. Additionally, certain nanozymes enable real-time monitoring *via* MRI or fluorescence imaging [116]. Clinical translation is progressing, evidenced by an ongoing Phase 1/2 trial (NCT05370508) evaluating SDT with aminolevulinic acid hydrochloride (SONALA-001) and MR-guided focused ultrasound. This study assesses safety, dosing, and efficacy to establish clinical viability [117].

Beyond conventional targeting, dual-targeting and multi-responsive nanoplatforms offer new solutions for GBM resistance. For instance, the approach involves a CD98hc-based bispecific antibody shuttle [118]. By targeting the highly expressed LAT1 amino acid transporter-rather than the traditional transferrin receptor (TfR)-this platform significantly elevates and prolongs cerebral drug concentrations. Murine studies demonstrate sustained therapeutic levels for up to 7 days. Furthermore, this system successfully delivers therapeutic antibodies and TrkB agonists, suggesting broader utility for neurological disorders [119].

Clearly, traditional drug delivery methods struggle to bypass these multifaceted biological barriers. To solve this, we need tools that can simultaneously navigate the BBB and precisely strike the molecular targets identified in Section 2. This necessity has driven the rapid evolution of the nanomedicine platforms discussed below.

## 4 TECHNOLOGICAL ADVANCES IN NANO DRUG DELIVERY SYSTEMS

### 4.1 Nanoparticles for Targeting Strategies in Glioblastoma

#### 4.1.1 Passive Targeting (EPR effect)

Passive targeting relies on the "enhanced permeability and retention effect" (EPR effect) of the abnormal vasculature of the tumour tissue, enabling passive enrichment of nanoparticles in GBM regions. Despite the increased vascular permeability of GBM, the heterogeneity of the BBB and the tumour microenvironment limits the full exploitation of the EPR effect [23, 120]. In recent years, studies have improved the passive targeting efficiency by optimising nanoparticle particle size, surface modification and combining physical means (*e.g*., ultrasound, permeability enhancers, *etc*.), but the overall efficacy is still limited, and therefore often combined with active targeting and other strategies [104]. Regarding the EPR effect, while it is well demonstrated in mouse models, glioblastoma (GBM) in humans typically grows beneath the surface with a more intact blood-brain barrier (BBB). This means that the passive accumulation effect of EPR alone is insufficient to enable nanoparticles to reach and accumulate around tumor cells responsible for recurrence. This discrepancy between mouse models and humans is one of the primary reasons why drug-loaded nanomaterials fail in clinical trials.

#### 4.1.2 Active Targeting (Ligand Modification)

Active targeting is achieved by modifying specific ligands (*e.g*., transferrin, Angiopep-2, RGD peptides, antibodies, *etc*.) on the surface of nanoparticles to achieve precise recognition and binding of highly expressed receptors on GBM cells or BBB endothelial cells, which facilitates nanomedicines to cross the BBB and enrich at the tumour site [111, 118]. For example, transferrin-modified liposomes and Angiopep-2-modified nanomedicines have been shown to significantly improve brain delivery efficiency and tumour targeting. This strategy significantly improves drug selectivity and therapeutic efficacy and has become the focus of current research on nanodelivery systems [109]. It is important to note that the binding efficiency observed *in vitro* often diminishes *in vivo* due to the formation of a 'protein corona.' Upon entering the bloodstream, plasma proteins rapidly adsorb onto the nanoparticle surface, potentially masking the targeting ligands (*e.g*., transferrin or RGD) [121]. This biological interaction can alter the nanoparticle's identity, directing it to the liver or spleen instead of the brain tumor, thereby reducing the intended targeting effect.

#### 4.1.3 Biomimetic Targeting

Bionanotargeting confers natural immune escape and tumour homing capabilities by encapsulating nanoparticles in cell membranes (*e.g*. leukocytes, stem cell membranes, tumour cell membranes, *etc*.). Biomimetic nanoparticles can effectively prolong *in vivo* circulation time and achieve active homing and targeted delivery of GBM using membrane surface molecules, which enhances delivery efficiency and biosafety [122, 123]. In addition, biogenic nanocarriers such as exosomes also show good potential for brain-targeted delivery and overcoming drug resistance [124].

#### 4.1.4 Stimulus Responsive Targeting

Stimulus-responsive nanoparticles enable precise drug release based on specific stimuli from the tumour microenvironment (*e.g*., acidic pH, high glutathione, proteases, *etc*.) or exogenous stimuli (*e.g*., ultrasound, magnetic field, light) [125]. This strategy enhances the selective release of drugs at the tumour site, reduces damage to normal tissues, and improves the safety and efficacy of treatment. For example, pH-responsive liposomes, redox-responsive polymer nanoparticles, and ultrasound/photoresponsive platforms have all made positive progress in GBM models [114].

#### 4.1.5 Combined-targeting Strategy

Co-targeting achieves multi-dimensional precision therapy by integrating multiple targeting mechanisms (*e.g*. dual ligand modification, active + stimulation response, *etc*.). This strategy can cover multiple tumour-associated receptors or microenvironmental features at the same time, which significantly enhances the delivery efficiency of nanomedicines and anti-drug resistance, and is an important development direction for future GBM nanotherapeutics [118, 126].

### 4.2 Various Nanodelivery Systems

#### 4.2.1 Liposome Drug Delivery Systems

Liposomes, as spherical vesicles formed by phospholipid bilayers, constitute the earliest clinically validated nano-delivery systems owing to their innate biocompatibility and adaptable surface characteristics. Recent progress has considerably expanded their therapeutic implementation in glioblastoma (GBM) management. Although conventional liposomal formulations encounter intrinsic limitations in crossing the blood-brain barrier (BBB), scientific efforts have developed advanced functionalization approaches to optimize targeting specificity. A key innovation encompasses transferrin receptor (TfR)-guided delivery mechanisms that dramatically increase liposomal accumulation within GBM tissues. This methodology was exemplified in a seminal investigation utilizing transferrin-conjugated cationic liposomes for dual delivery of temozolomide (TMZ) and MGMT siRNA. Although the combinatorial platform reduced tumor volume by approximately 78% and extended survival by 2.5-fold in murine models, it is important to note that these results were obtained in subcutaneous or orthotopic xenografts. These models often fail to fully replicate the complex immune microenvironment and intact blood-brain barrier found in human patients, potentially leading to an overestimation of clinical efficacy [120].

pH-responsive liposomes constitute another significant advancement in this field. Given the weakly acidic microenvironment of GBM (pH 6.5-6.8), researchers have engineered liposomes capable of undergoing structural transformations under acidic conditions to achieve precise drug release at tumor sites. For instance, liposomes incorporating the pH-sensitive lipid DOPE transition from a lamellar to a hexagonal phase structure in acidic environments, thereby facilitating content release and augmenting TMZ cytotoxicity [93]. Recent developments have further yielded multifunctional liposomal systems, such as photothermal-chemotherapy synergistic liposomes. These systems generate thermal effects *via* near-infrared light excitation while concurrently releasing TMZ, enabling dual therapeutic action. Additionally, lipid nanoparticles (LNPs) have been successfully adapted for mRNA delivery, establishing a novel platform for GBM gene therapy [127] (Table **1**).

#### 4.2.2 Polymer Drug Delivery Systems

Polymer-based nano-delivery systems have garnered significant research interest owing to their tunable physicochemical properties and versatile functionalization capabilities. Commonly employed polymeric materials include poly (lactic-co-glycolic acid) (PLGA), polyethylene glycol (PEG), and polyethyleneimine (PEI). PLGA, an FDA-approved biodegradable polymer, exhibits excellent biocompatibility. Investigators have engineered PLGA nanoparticles for the co-delivery of temozolomide (TMZ) and cedarbenamine (an HDAC inhibitor), significantly amplifying TMZ's antitumor efficacy through MGMT suppression *via* epigenetic modulation. Furthermore, a separate study leveraged PLGA-PEG nanoparticles to simultaneously deliver TMZ and miR-221/222 inhibitors, successfully reversing TMZ resistance in GBM cells by achieving approximately 75% reduction in IC50 values [133]. However, the "sudden release phenomenon" remains a pressing challenge. The immediate release of large quantities of active ingredients after administration may trigger systemic toxic reactions. Moreover, in large-scale production processes compliant with GMP standards, maintaining consistency in molecular weight and degradation rates continues to pose a significant barrier to industrial application.

Dendrimeric polymers (*e.g*., polyamidoamine [PAMAM]) serve as exemplary gene delivery vectors due to their hyperbranched architecture and profusion of surface functional groups. Research demonstrates that engineered PAMAM dendrimers efficiently deliver MGMT siRNA, markedly suppressing MGMT expression while potentiating TMZ sensitivity. Nevertheless, the inherent cytotoxicity of higher-generation PAMAM constrains its clinical utility, prompting the development of surface modification strategies such as PEGylation or amino acid functionalization to mitigate biocompatibility concerns [134].

Stimuli-responsive polymeric nanosystems have emerged as a focus of intensive research in targeted therapeutics. Redox-sensitive polymers incorporating disulfide bonds undergo selective cleavage within tumor microenvironments characterized by elevated glutathione concentrations, enabling spatiotemporally controlled drug release. Concurrently, thermoresponsive polymers (*e.g*., poly(N-isopropylacrylamide) [PNIPAM]) exhibit reversible phase transitions modulated by physiological or externally applied thermal stimuli, thereby regulating therapeutic payload delivery. These intelligent, responsive mechanisms substantially augment treatment specificity while minimizing off-target effects [135] (Table **2**).

#### 4.2.3 Inorganic Drug Delivery Systems

Inorganic nanomaterials such as gold nanoparticles (AuNPs), mesoporous silica, and iron oxide nanoparticles exhibit distinctive physicochemical properties and versatile functionality. AuNPs demonstrate precise control over size, morphology, and surface chemistry, while offering facile functionalization capabilities. Furthermore, AuNPs serve as effective agents for photothermal therapy, wherein near-infrared light irradiation generates localized hyperthermia that synergistically enhances chemotherapeutic efficacy to induce tumor cell ablation [139, 140].

Mesoporous silica nanoparticles (MSNs) exhibit exceptionally high surface area, precisely tunable pore dimensions, and favorable biocompatibility. Research demonstrates that folic acid-functionalized MSNs facilitate targeted co-delivery of TMZ and MGMT promoter-methylating agents, effectively suppressing MGMT expression *via* epigenetic modulation to counteract TMZ resistance. In a complementary approach, investigators employed MSNs to concurrently deliver TMZ and miR-221 inhibitors, demonstrating enhanced antitumor efficacy through dual inhibition of DNA repair mechanisms and cell cycle progression pathways, as evidenced by a 75% reduction in IC50 values compared to monotherapy controls [141]. While gold and silica nanoparticles offer precise control over size and functionalization, their non-biodegradability poses long-term safety concerns. Unlike lipids or polymers, inorganic carriers may accumulate in the liver or spleen, leading to potential chronic toxicity. While their therapeutic efficacy is promising, their regulatory path to approval is likely to be more arduous compared to organic counterparts.

Superparamagnetic iron oxide nanoparticles (SPIONs) demonstrate multifunctional therapeutic potential, serving not only as drug delivery vehicles but also as contrast agents for magnetic resonance imaging (MRI), thereby enabling integrated diagnostic and therapeutic applications. Furthermore, under alternating magnetic fields, SPIONs generate localized magnetothermal effects that synergize with chemotherapeutic agents to induce tumor cell apoptosis [142] (Table **3**).

### 4.3 Biogenic Nanoscale Delivery Platforms

Biologically derived nano-delivery systems, such as exosomes and cell membrane vesicles, exhibit inherent biocompatibility and intrinsic targeting capabilities, positioning them as a major focus of contemporary biomedical research. Exosomes, nanoscale extracellular vesicles (30-150 nm) secreted by cells, transport biologically active cargo (proteins, nucleic acids, and signaling molecules) critical for intercellular communication. Pioneering studies validate the therapeutic potential of exosomal platforms, including bevacizumab (BEV)-loaded exosomes that efficiently traverse the blood-brain barrier (BBB) to suppress glioblastoma (GBM) progression by inhibiting tumor angiogenesis [115]. Furthermore, exosomes encapsulating non-coding RNAs (ncRNAs) demonstrate significant utility in circumventing TMZ chemoresistance in GBM, highlighting their translational potential for clinical oncology applications [146].

Cell membrane vesicles represent another category of biologically derived nanocarriers. Researchers have engineered biomimetic nanovehicles by encapsulating poly(lactic-co-glycolic acid) (PLGA) nanoparticles within macrophage-derived membranes. This design leverages innate "self-marking" mechanisms to evade immune clearance, thereby prolonging systemic circulation duration and enhancing tumor-targeting specificity. Furthermore, nanoparticles functionalized with tumor cell membranes exhibit augmented cellular uptake through homotypic targeting mechanisms, significantly improving therapeutic efficacy compared to conventional delivery systems [122]. While these biofilms demonstrate significant advantages *in vivo*, their standardized production faces inherent challenges due to individual variations in extracellular vesicles. Unlike synthetic liposomes or inorganic nanoparticles mentioned earlier, cell-derived exosomes often exhibit batch-to-batch compositional differences. This raises critical questions about the suitability of such biofilms for direct human application, requiring further evaluation.

In recent years, virus-like nanoparticles (including lyssaviruses, virus-like particles [VLPs], phages, *etc*.) have demonstrated unique advantages as a new generation of biologically-derived nano-delivery platforms for the treatment of CNS diseases, especially gliomas. Compared with traditional synthetic nanocarriers (*e.g*., polymer nanoparticles, liposomes, *etc*.), virus-like nanomaterials have a highly homogeneous particle size distribution, excellent biocompatibility, degradability, and natural cell targeting ability [147, 148]. In addition, the protein shell structure of viral nanoparticles can be genetically engineered or chemically modified to achieve surface functionalisation for efficient loading and precise delivery of a wide range of active molecules such as drugs, nucleic acids, proteins, *etc*.

VLPs, as viral capsids without genetic material, combine the structural advantages of viruses with biosafety, and have been widely used in the fields of drug delivery, gene therapy, and vaccine development. Systematically evaluated the use of three different morphologies of VLPs (MS2 sphere, Tobacco mosaic virus [TMV] disc, and nano phage rod) as a DOX delivery vehicle in GBM models [149]. It was found that VLP-DOX was able to efficiently enter U87-MG glioma cells and release the drug, and after convection-enhanced delivery (CED) to mouse intracerebral tumours, the survival of mice was significantly prolonged in the TMV disc-shaped and MS2 spherical VLP-DOX groups, showing the influence of the VLP morphology on the effectiveness of the drug delivery. Notably, VLP-DOX was administered at a much lower dose than the conventional intravenous dose, suggesting its potential for efficient drug delivery while reducing systemic toxicity [147, 150].

In addition to VLPs, Oncolytic Viruses (OVs), as another important class of viral nanoplatforms, have attracted much attention in recent years in preclinical and clinical studies of brain tumours, such as GBM, due to their ability to selectively replicate in tumour cells and induce tumour cell lysis, as well as activate anti-tumour immune responses. For example, genetically engineered adenoviruses such as DNX-2401 (Delta-24-RGD) have entered the clinical trial stage, showing good safety and some therapeutic effects. Oncolytic viruses can also be used as delivery platforms to carry suicide genes, immunomodulatory factors, or chemotherapeutic drugs to achieve multimodal synergistic anti-tumour effects [151-153].

Phages and their derived nanoparticles also have good biosafety and engineering properties. ff phages (*e.g*. M13, fd, *etc*.) can not only be screened for specific tumour-targeting peptides by surface display technology, but also be chemically modified to achieve highly efficient delivery of drugs and nucleic acids. Studies have shown that phage nanoparticles can traverse the BBB *via* nasal or CED routes, *etc*., which provides a new idea for targeted therapy of brain diseases [154, 155] (Table **4**).

## 5 NANOCARRIERS AND THEIR ROLE IN GENE THERAPY

Currently, gene editing technologies such as siRNA and CRISPR-Cas9 have dramatically revolutionised therapeutic strategies for GBM resistance to TMZ. siRNA can target silencing of anti-apoptotic proteins such as MGMT or Bcl-2, thereby directly weakening the chemotherapy resistance of tumour cells; CRISPR-Cas9 enables permanent knockdown of resistance-related genes (*e.g*., mismatch repair components), and even epigenetic reprogramming of tumour stem cells. However, single-nucleic acid drugs generally face three major obstacles during clinical translation: 1) rapid degradation by *in vivo* enzymes, 2) difficulty in efficiently traversing the BBB, and 3) off-target risk. It is worth noting that only by combining these gene editing tools with nanoscale carriers (*e.g*., liposomes, exosomes, *etc*.) can we effectively achieve *in vivo* homeostatic protection and precise delivery, thus truly realising "nanotherapeutics" [126, 159, 160]. Although nanoplatforms have successfully crossed the blood-brain barrier (BBB), gene therapy still faces a critical hurdle: the intracellular barrier. The challenge of being engulfed by lysosomes remains a major bottleneck. Studies show that less than 2% of the payload in lipid nanoparticles escapes endosomes to reach the cytoplasm or nucleus, severely limiting actual gene-editing efficiency [161]. Moreover, unlike transient drugs, CRISPR-Cas9 induces permanent genomic alterations. Any unintended "targeted" mutations or chromosomal rearrangements in non-tumor brain cells pose long-term safety risks that transient siRNA therapies cannot address.

### 5.1 Nanocarriers: the Key to Precise Gene Therapy Delivery

Nanocarriers, such as cationic polymer-modified LNPs and functionalized peptide-modified exosomes, enable efficient delivery of therapeutic molecules into the brain by 1) stably encapsulating nucleic acid drugs, such as siRNA/CRISPR, and preventing them from degradation; and 2) enabling efficient delivery of therapeutic molecules into the brain through the transBBB transcytophagy pathway mediated by transferrin receptor or LDL receptor. The stimulus-responsive nanoplatforms developed in recent years can even precisely release gene editing tools in the tumour microenvironment (*e.g*. acidic environment), achieving therapeutic controllability in time and space and greatly enhancing target specificity.

### 5.2 A New Paradigm for Synergistic Therapy with Nanodelivery Systems

The nanocarriers also enable synergistic encapsulation and delivery of TMZ with e siRNA and CRISPR. These multifunctional nanocarriers can simultaneously block resistance pathways (*e.g*. MGMT inhibition) and enhance TMZ-induced DNA damage, resulting in more significant synergistic therapeutic effects [162]. This integrated strategy not only breaks the compensatory effects of multiple resistance pathways but also significantly reduces systemic immunogenicity, further highlighting the transformative potential of nanomedicine combined with molecular precision therapy.

### 5.3 Advantages of the "Gene Drug + Nanocarrier" Combination Strategy

It should be clear that siRNA or CRISPR-Cas9 therapies alone are only gene therapies, and the realisation of their true nanoscale precision delivery relies on nanocarriers. This combined strategy radically enhances therapeutic efficacy by targeting key resistance pathways such as MGMT-mediated DNA repair and Bcl-2 anti-apoptotic signalling, while significantly reducing the risk of off-target and toxic side effects (Table **5**). The controlled release capability of the nanodelivery system provides a superior therapeutic index and safety for clinical translation.

## 6 BARRIERS TO CLINICAL TRANSLATION AND REGULATORY PERSPECTIVES

The transition of nanomedicines from preclinical success to clinical application is severely impeded by a high rate of translational failure, primarily driven by the discordance between the nanoparticle's engineered physicochemical properties and its altered surface characteristics acquired within the biological environment [174]. Upon intravenous administration, nanoparticles undergo an immediate surface remodeling, adsorbing plasma proteins to form a dynamic "protein corona" composed of a tightly bound "hard" layer and a rapidly exchanging "soft" layer [175, 176]. This corona effectively masks engineered surface ligands (*e.g*., transferrin or angiopep-2), neutralizing their ability to engage specific receptors on the BBB [177, 178]. Crucially, the composition of this corona is dictated by the nanoparticle's surface chemistry and morphology; for instance, cationic liposomes often attract negatively charged opsonins like immunoglobulins and complement proteins, which trigger rapid immune recognition rather than the intended BBB transcytosis [179, 180].

Furthermore, systemic clearance mechanisms and physiological barriers in humans far exceed those in rodent models, leading to poor predictive validity of preclinical data [174]. The protein corona not only alters targeting capabilities but also significantly impacts the physicochemical stability of carriers; for lipid-based systems, protein adsorption can induce membrane restructuring and premature leakage of hydrophilic payloads like temozolomide before they reach the intracranial target [180-182]. Consequently, the vast majority of administered doses are sequestered by the mononuclear phagocyte system (MPS) in the liver and spleen, with often less than 1% accumulating in the brain tumor [183]. While strategies such as "biomimetic camouflage" or recruiting endogenous transport proteins (*e.g*., Apolipoprotein E) into the corona have been proposed to enhance brain delivery, controlling these interactions in the complex human proteome remains elusive [184, 185].

Finally, the unpredictability of these bio-nano interactions poses monumental challenges for Good Manufacturing Practice (GMP) scalability and regulatory approval [186, 187]. The formation of the protein corona is highly sensitive to patient-specific factors (*e.g*., disease state, plasma proteome variations), meaning the same nanomedicine may exhibit different pharmacokinetics and toxicity profiles in different patients [188-190]. This variability complicates the establishment of standardized safety profiles required by agencies like the FDA, particularly for inorganic carriers where long-term neurotoxicity and bio-persistence are major concerns [186, 191, 192]. Therefore, future translation demands not only reproducible large-scale manufacturing but also advanced "safety-by-design" strategies that account for the dynamic evolution of nanoparticles within the biological environment.

## CONCLUSION AND FUTURE PERSPECTIVES

The advancement of nano-engineered drug delivery systems has revolutionized the targeting of TMZ resistance in GBM, enabling precise intervention against intratumoral heterogeneity and molecular evasion mechanisms. Through the co-delivery of chemotherapeutic agents with gene-editing tools (*e.g*., CRISPR-Cas9) or tumor microenvironment-modulating molecules *via* multifunctional nanocarriers, researchers have synergistically disrupted resistance pathways-such as concurrent suppression of MGMT expression and potentiation of DNA damage response pathways-while significantly mitigating systemic toxicity [130]. Innovations in biomimetic architectures (*e.g*., bone marrow-derived mesenchymal stem cell vesicles) and stimuli-responsive platforms (*e.g*., glutathione-triggered release mechanisms) have further overcome critical barriers, including efficient blood-brain barrier penetration and precise spatiotemporal control of therapeutic payload distribution [130, 170].

Ultimately, addressing TMZ resistance demands a paradigm shift from monotherapeutic approaches to multifunctional nano-theranostic platforms that orchestrate multimodal therapeutic modalities-chemotherapy, epigenetic regulation, and immunomodulation-within a single-system architecture. By converging molecular pathogenesis insights with advanced biomaterial engineering to target unmet clinical challenges, this convergence of multidisciplinary expertise may catalyze the evolution of GBM management from a terminal diagnosis to a chronically controllable condition with extended progression-free survival. How to increase the yield of nanomaterials and how to verify their efficacy in the human body are also urgent problems to be solved before nanomaterials can be applied in clinical practice.

## Figures and Tables

**Fig. (1):**
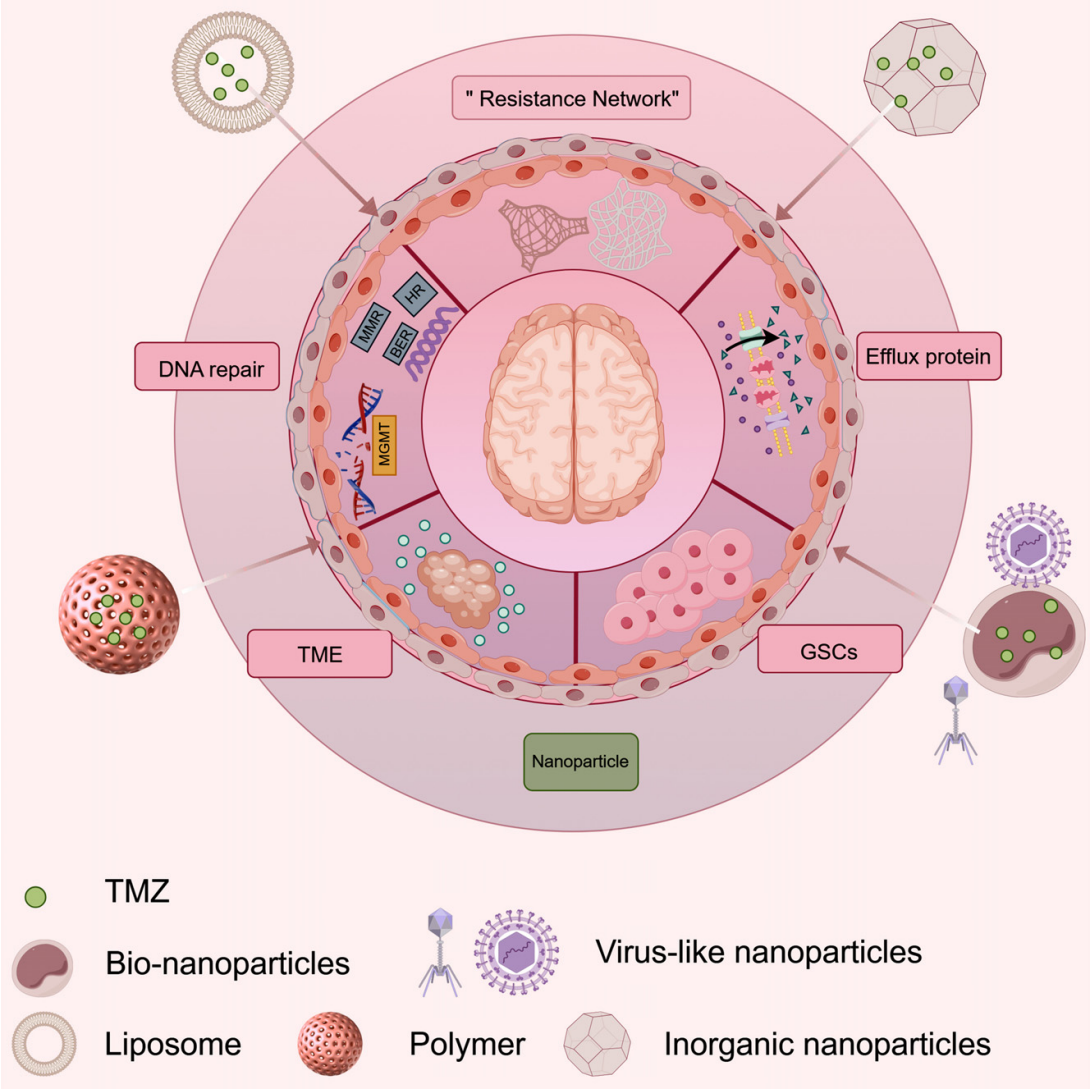
TMZ resistance and nanodelivery systems. By figdraw.com.

**Fig. (2):**
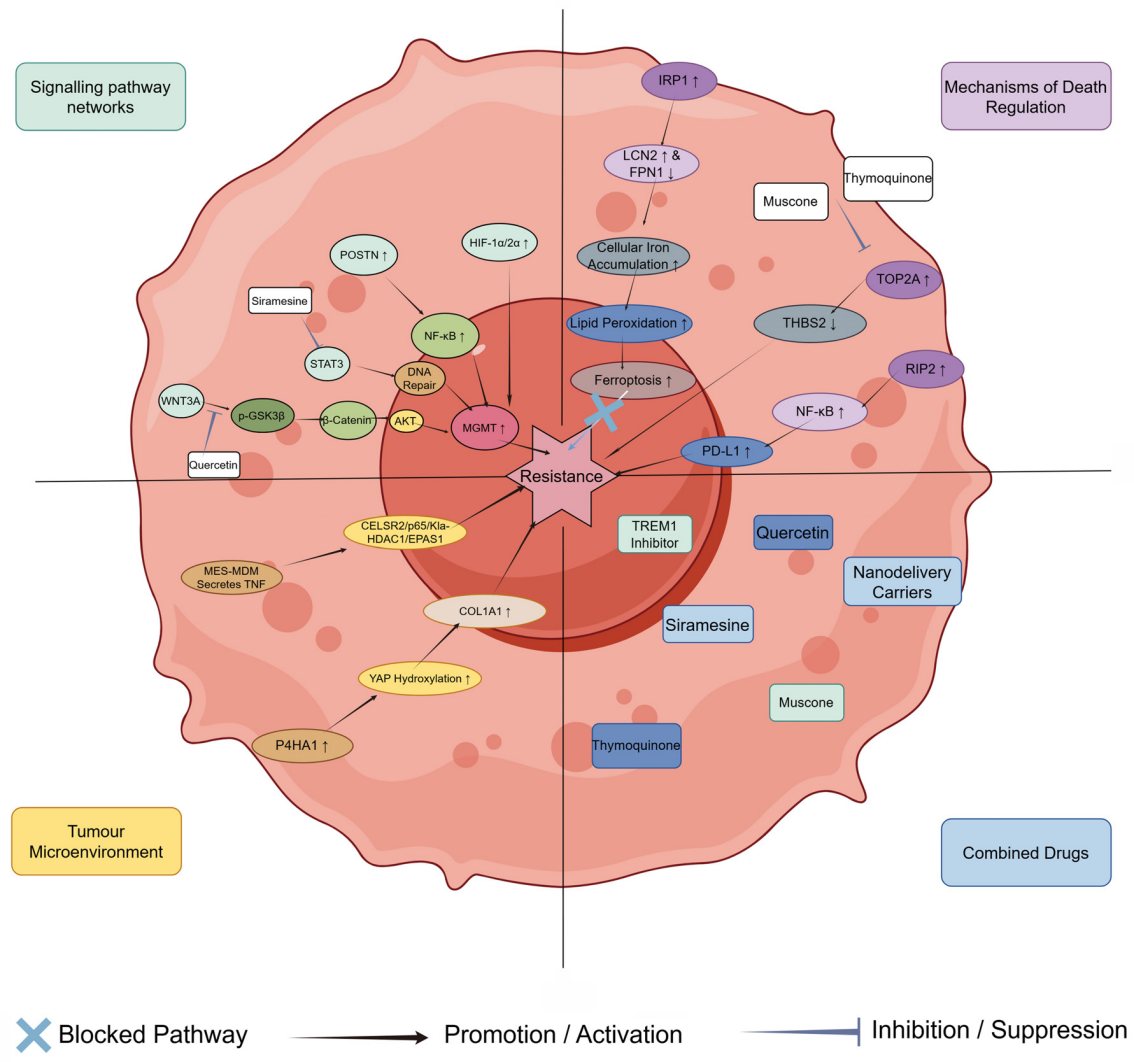
Multidimensional network mechanism map of TMZ resistance in glioblastoma. The diagram illustrates the complex interplay between molecular signaling and the tumor microenvironment driving resistance. Signaling Networks (Left): Key pathways such as WNT/*β*-Catenin and PI3K/AKT drive *MGMT* expression and DNA repair. Death Regulation (Right): Dysregulation of ferroptosis and apoptosis mechanisms. Therapeutic Interventions: Small molecules and nanodelivery systems designed to target specific nodes to reverse resistance. Abbreviations: TMZ, temozolomide; *MGMT*, O6-methylguanine-DNA methyltransferase. By figdraw.com.

**Fig. (3):**
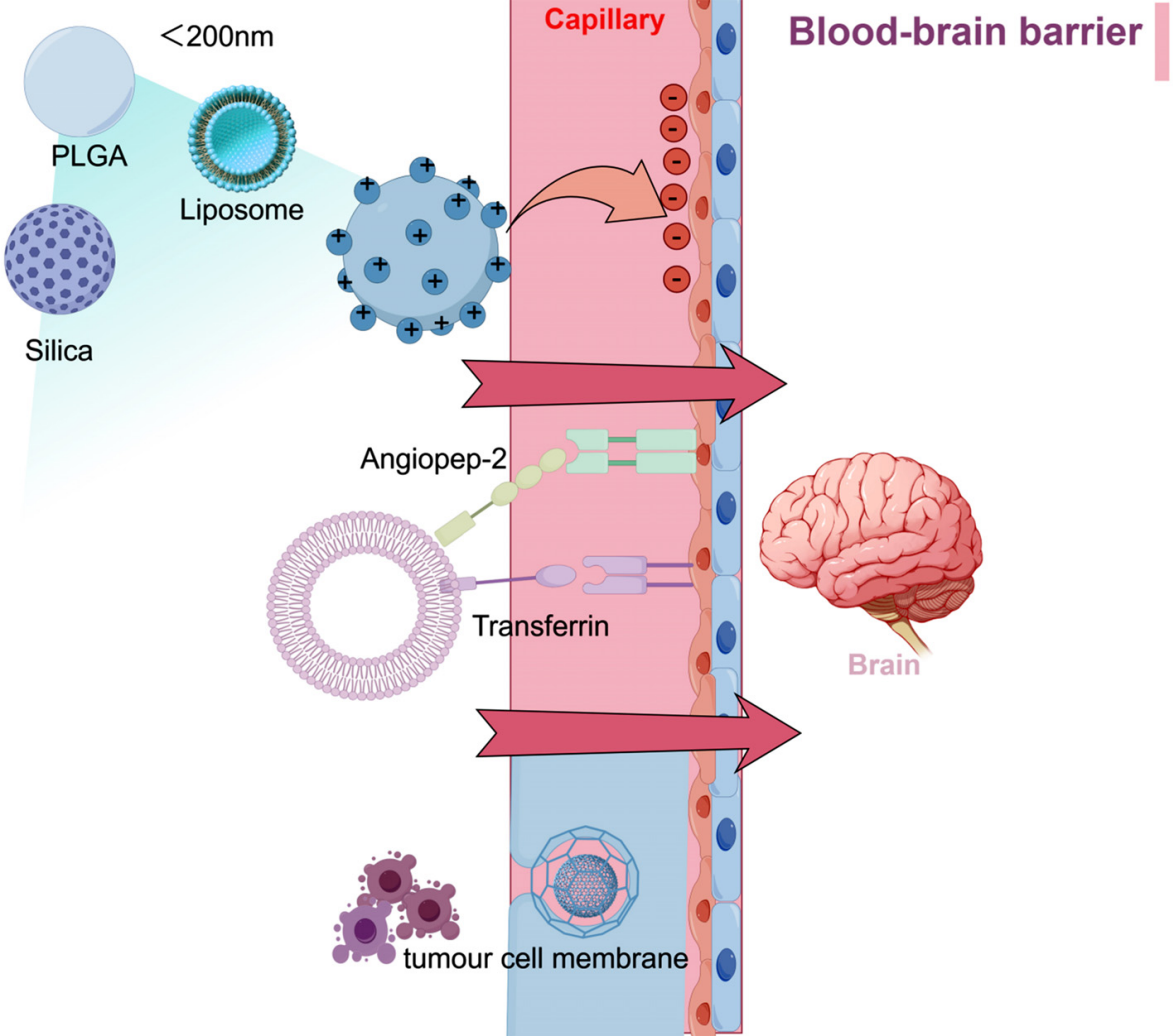
Nanoparticles cross the BBB. By figdraw.com.

**Table 1:** Summary of functionalized liposomal formulations engineered to overcome TMZ resistance in glioblastoma.

**Liposome Type**	**Retouching Strategy**	**Delivery of Drugs**	**Key Benefits**
Multifunctional liposomeLPO@cu2se-gox	Cu_2_Se	Glucose oxidase (GOx)	Enhanced drug delivery efficiency, self-supplied H_2_O_2_ production, induced iron death, improved immune response [128]
Thermosensitive liposomes, TSL anti-EphA3-TMZ16e-GNPs-TSL	Anti-EphA3 antibody modification, gold nanoparticles (GNPs), thermosensitive design	TMZ derivative TMZ16e,Gold nanoparticles (GNPs)	Strong targeting, photothermal synergistic therapy, temperature-controlled release, improved drug bioavailability [129]
Polymer-locked fusion liposomes (Plofsome)	Four-armed polyvinyl alcohol-catechol (4-arm PEG-oDP), ROSFissile chain	siRNA, CRISPR-Cas9 RNP	Strong targeting, effective crossing of the BBB, and reduced drug resistance [130]
Polyethylene glycol (PEG)-modified liposomes T + BG@PEG-LP	PEG-modified, dual-drug co-loading	TMZ, O6-Benzylguanine (O6-BG)	Enhanced cellular uptake, improved drug release properties, synergistic anti-tumor effects [131]
Polyvinyl alcohol-modified thermosensitive liposomesT + E@LPs-cRGD+GelMA	GelMA (gelatin methacrylamide) hydrogel, cyclic RGD peptide (cRGD) modification	TMZ, Erastin (ERA)	Strong targeting, sustained release properties, overcoming drug resistance, overcoming drug resistance, immunomodulatory effects [132]
Modified liposomes (Bo-Gas-LP)	(R)-(+)-bornylamine	TMZ	Enhanced drug sensitivity, blocking astrocyte-glioblast interaction, good biocompatibility, and stability [125]

The table details specific surface modification strategies, co-delivered therapeutic agents, and key biological benefits such as enhanced BBB penetration.

**Table 2:** Overview of polymeric nanocarrier systems utilized to target specific resistance mechanisms.

**Polymer Type**	**Delivery of Drugs**	**Response Mechanism**	**Key Benefits**
Poly(d,l-lactic-co-ethanolic acid)-monomethoxypoly(vinyl alcohol)(PLGA-mPEG) TMZ/LIG-NPs	TMZ, Lisdexamfetamine (LIG)	Inhibition of P-glycoprotein and tight junction proteins	Synergizing anti-tumor effects and overcoming tumor resistance [136]
Glycosylated and cascade-responsive nanoparticle (GCNP)	CRISPR/Cas9	cascade response	Safety and efficacy of gene editing, significantly improved anti-tumor effects, improved drug sensitivity [137]
Polymeric poly(d,l-lactic-co-glycolic) acid (PLGA)	TMZ	Nasal-brain delivery mechanism	Antitumor and antiangiogenic effects, clinical translational potential, homologous targeting [138]
Modified zein, by chemical modification with polyethylene glycol (PEG) and Angiopep-2 (ANG2)	Docetaxel, DTX	Through the low-density lipoprotein receptor (LDLR)	Targeted delivery, biocompatibility, and biodegradability improved drug bioavailability [110]

The data highlights various polymer types (*e.g*., PLGA), their stimulus-responsive properties or targeting modifications, and efficacy in delivering chemotherapeutic and gene-editing payloads.

**Table 3:** Comparative analysis of inorganic nanoparticle platforms (Gold, Silica, Iron Oxide) applied in glioblastoma therapy.

**Inorganic Nanomaterials**	**Retouching Strategy**	**Delivery of Drugs**	**Multi-functional Features**
Iron oxide nanoparticles	Graphene oxide and chitosan (CS) binding	TMZ	Magnetic response, pH sensitivity, drug sensitivity [143]
Mesoporous silica nanoparticles (C8-MSN)	Functionalization of C8 silanes, modification of poly(ethylene glycol) (PEG), and organic quaternary ammonium salts	DTX	Targeted delivery, controlled release, biocompatibility, enhanced cellular uptake [144]
Radioactive gold nanoparticles (RGNP)	Modified, non-radioactive precursor synthesis by poly(ethylene glycol) (PEG6000)	TMZ	Radiation therapy, synergistic treatment effects, targeted delivery, drug sensitivity [124]
Gold nanoparticles (AuNPs)	Transferrin-like peptides (TFP) binding for functionalization, Polyethylene glycol (PEG) coating	TMZ	Targeted delivery, enhanced drug penetration, cellular uptake, and cytotoxicity, and drug sensitivity [145]

The table outlines their physicochemical properties, functionalization for targeted delivery, and multifunctional capabilities, including dual-modal imaging and photothermal therapy integration.

**Table 4:** Biogenic nanoscale delivery platforms are utilized for CNS drug delivery.

**Biosourced Nanosystems**	**Source**	**Delivery of Drugs**	**Key Benefits**
Exosomes	glioblastoma cell	TMZ and EPZ015666	Cell proliferation inhibition, targeting, and low toxicity [156]
Exosomes	Human glioblastoma cells (U-87 MG)	Celastrol (Cel)	High drug loading efficiency, good biocompatibility, effective tumor inhibition [157]
Exosomes	Adipose tissue-derived mesenchymal stem cells (ADSCs)	TMZ and quercetin (Qct)	Enhanced targeting, improved drug bioavailability, synergistic therapeutic effect, pH-sensitive release [158]
Engineered artificial drug-carrying vesicles (EAVs), ANG-TRP-PK1@EAVs	HEK293T cells	DOX	High yield, effective BBB penetration, enhanced anti-tumor effects, good biocompatibility, targeting [123]
Virus-like particles (VLPs, MS2)	Bacterial recombinant expression	DOX	Uniform size, high biocompatibility, efficient BBB penetration, reduced systemic toxicity, prolonged survival in the GBM model [149]
Virus-like particles (VLPs, phage nanorod)	Phage (Ff bacteriophage)	DOX	Rod-shaped, high drug loading, shape-dependent BBB transport [149]
Oncolytic virus (adenovirus, HSV, *etc*.)	Engineered viral strains	Gene therapy, immunomodulators, cytotoxic agents	Tumor-selective replication and lysis, breaks resistance, immune activation, and can cross the BBB [154]
Bacteriophage nanocarriers	Recombinant filamentous bacteriophage	Peptides, nucleic acids, drugs	Easy surface modification and targeting, ability to cross the BBB, robust stability [147]

The table categorizes carriers by biological source (*e.g*., exosomes, virus-like particles) and describes their intrinsic advantages, such as biocompatibility, immune evasion, and natural blood-brain barrier crossing abilities.

**Table 5:** Synergistic strategies combining gene-editing technologies (siRNA, CRISPR-Cas9) with nanocarriers to reverse temozolomide resistance.

**Gene Target**	**Delivery of Contents**	**Nanomaterials**	**Key Benefits**
MGMT	siRNA	Nucleic acid framework (FNN-siMGMT-ANG)	Significantly increased sensitivity to TMZ and decreased proliferative capacity [163]
MDK	siRNA, CRISPR-Cas9	Liposome	Significantly increased sensitivity to TMZ and decreased proliferative capacity [130]
STAT3	siRNA	Biofilms: Cancer Cell Membranes	TMZ increased drug sensitivity, improved BBB penetration, induced apoptosis of tumour cells, effectively inhibited tumour proliferation, and significantly prolonged the survival of mice [164]
MGMT	CRISPR-Cas9	Lipid-polymer hybrid nanoparticles (LPHNs-cRGD)	Increased TMZ sensitivity, inhibition of tumour growth, and significantly prolonged survival in mice [165]
Gli1	siRNA	Polyethyleneimine-Spherical Nucleic Acid Nanoparticles (PEI-SNA)	Increased TMZ sensitivity, inhibited tumour growth, reduced tumour stem cell self-renewal capacity, induced apoptosis [166]
Galectin-1	siRNA	Chitosan nanoparticles	Increased chemosensitivity, improved immunotherapy efficacy, inhibition of tumour cell proliferation and inhibition of M2 macrophage polarization [167]
MGMT	siRNA	Iron oxide nanoparticles	Elevated TMZ sensitivity, induction of tumour stem cell apoptosis, low toxicity, and good biocompatibility [168]
MGMT	siRNA	PEG2000-Poly(Met) nanoparticles	Increased TMZ sensitivity, inhibited tumour cell proliferation, induced tumour cell apoptosis, and enhanced radiotherapy sensitivity [169]
STAT3	siRNA	Bone marrow mesenchymal stem cell (BMSC)-derived exosomes	Elevated TMZ sensitivity induces tumour cell apoptosis and prolongs mouse survival [170]
YY1	siRNA	Exosome	Increased sensitivity to TMZ and radiotherapy, reversal of treatment resistance, inhibition of tumour cell proliferation, and no significant organ toxicity [171]
MALAT1	siRNA	Cationic liposome	Increased TMZ sensitivity, inhibition of tumour proliferation and migration, induction of tumour cell apoptosis [172]
EGFR	siRNA	Polymer nanoparticles	Increased TMZ sensitivity and inhibition of tumour growth without significant organ toxicity [173]

The table correlates specific genetic targets (*e.g*., *MGMT, STAT3*) with corresponding delivery vectors and summarizes the therapeutic impact on tumor sensitivity.

## LIST OF ABBREVIATIONS

ABC: ATP-binding Cassette
ADD3: Adenylate Cyclase-associated Protein 3
ANG2: Angiopep-2
AuNPs: Gold Nanoparticles
BBB: Blood-brain Barrier
BCRP: Breast Cancer Resistance Protein
BER: Base Excision Repair
BMECs: Brain Microvascular Endothelial Cells
BTB: Blood-tumor Barrier
CBTRUS: Central Brain Tumor Registry of the United States
CCN1: Cell Communication Network Factor 1
CD98hc: CD98 Heavy Chain
CDT: Chemodynamic Therapy
Ce6: Chlorin e6
CMC: Chemistry, Manufacturing, and Controls
CNS: Central Nervous System
CRISPR: Clustered Regularly Interspaced Short Palindromic Repeats
Cx43: Connexin-43
DDR: DNA Damage Repair
DOX: Doxorubicin
DTX: Docetaxel
EPR: Enhanced Permeability and Retention
FUS: Focused Ultrasound
GAP-43: Growth-associated Protein 43
GBM: Glioblastoma Multiforme
GMP: Good Manufacturing Practice
GSCs: Glioma Stem Cells
HDAC: Histone Deacetylase
HR: Homologous Recombination
IC50: Half Maximal Inhibitory Concentration
ICWs: Intercellular Calcium Waves
IL-13Rα2: Interleukin-13 Receptor alpha 2
LiPTD NPs: Lithium Phthalocyanine-based Therapeutic Dendrimer Nanoparticles
LRP1: Low-density Lipoprotein Receptor-related Protein 1
MBs: Microbubbles
MGMT: O6-methylguanine-DNA Methyltransferase
MMR: Mismatch Repair
MPS: Mononuclear Phagocyte System
MRI: Magnetic Resonance Imaging
MRP1: Multidrug Resistance-associated Protein 1
MSNs: Mesoporous Silica Nanoparticles
MTA: Microtubule-targeting Agent
MTIC: 5-(3-methyltriazin-1-yl) imidazole-4-carboxamide
NER: Nucleotide Excision Repair
NVU: Neurovascular Unit
OS: Overall Survival
OVs: Oncolytic Viruses
PAMAM: Polyamidoamine
PEG: Polyethylene Glycol
PEI: Polyethyleneimine
PFS: Progression-free Survival
P-gp: P-glycoprotein
PLGA: Poly(lactic-co-glycolic Acid)
PTM: Post-translational Modifications
RMT: Receptor-mediated Transcytosis
ROS: Reactive Oxygen Species
SDT: Sonodynamic Therapy
siRNA: Small-interfering RNA
SPIONs: Superparamagnetic Iron Oxide Nanoparticles
TfR: Transferrin Receptor
TME: Tumor Microenvironment
TMs: Tumor Microtubes
TMZ: Temozolomide
TNF: Tumor Necrosis Factor
TTC: Tumor-tumor Connectivity
VEGF: Vascular Endothelial Growth Factor
VLPs: Virus-like Particles
ZO-1/ZO-2: Zonula Occludens-1/2
